# Functional cure with new antiviral therapy for hepatitis B virus: a systematic review and meta-analysis

**DOI:** 10.1007/s12072-025-10823-5

**Published:** 2025-06-18

**Authors:** Jing Chen, Dong Ji, Jidong Jia, Hui Zhuang, Xinxin Zhang, Fu-Sheng Wang, Wenhong Zhang, Xiaoguang Dou, Tawesak Tanwandee, Shiv Kumar Sarin, Rakhi Maiwall, Manoj Kumar, George Boon-Bee Goh, Hasmik Ghazinyan, Anuchit Chutaputti, Pei-Jer Chen, Hong You, Ming-Lung Yu, Jacob George, Masao Omata, Gui-qiang Wang, George Lau, APASL Viral Elimination Taskforce

**Affiliations:** 1 School of Public Health and Primary Care, Faculty of Medicine, Chinese University of Hong Kong, Hong Kong SAR, China; 2 Senior Department of Hepatology, Fifth Medical Center of Chinese, PLA General Hospital, Beijing, 100039 China; 3 Liver Research Center, Beijing Friendship Hospital, Capital Medical University, Beijing, China; 4 Department of Microbiology and Centre for Infectious Diseases, Peking University Health Science Centre, Beijing, China; 5 Research Laboratory of Clinical Virology, Ruijin Hospital, Shanghai Jiao Tong University School of Medicine, No.197 Ruijin 2nd Road, Shanghai, 200025 China; 6 Treatment and Research Center for Infectious Diseases, The Fifth Medical Center of PLA General Hospital, National Clinical Research Center for Infectious Diseases, Beijing, 100039 China; 7 Department of Infectious Diseases, Shanghai Key Laboratory of Infectious Diseases and Biosafety Emergency Response, National Medical Center for Infectious Diseases, Huashan Hospital, Fudan University, Shanghai, 200040 China; 8 Department of Infectious Diseases, Shengjing Hospital of China Medical University, Shenyang, 110000 China; 9 Division of Gastroenterology, Department of Medicine, Faculty of Medicine Siriraj Hospital, Mahidol University, Salaya, Thailand; 10 Department of Hepatology, Institute of Liver and Biliary Sciences, New Delhi, India; 11 Department of Gastroenterology & Hepatology, Duke-NUS Graduate Medical School, Singapore General Hospital, Singapore, Singapore; 12 Gastroenterology and Hepatology Service, Yerevan Medical Scientific Center, Yerevan, Armenia; 13 Section of Digestive and Liver Diseases, Department of Medicine, Phramongkutklao Hospital, Bangkok, Thailand; 14 Graduate Institute of Clinical Medicine, National Taiwan University, Taipei, Taiwan; 15 Beijing Friendship Hospital, Capital Medical University, Beijing, China; 16 Hepatobiliary Division, Department of Internal Medicine, Kaohsiung Medical University Hospital, Center of Hepatitis Research, College of Medicine and Center for Liquid Biopsy and Cohort Research, Kaohsiung Medical University, Kaohsiung, Taiwan; 17 Storr Liver Centre, The Westmead Institute for Medical Research, Westmead Hospital and University of Sydney, Sydney, NSW Australia; 18 Yamanashi Hospitals (Central and Kita) Organization, Kofu-shi, Yamanashi, Japan; 19 Department of Infectious Disease, Center for Liver Disease, Peking University First Hospital, Beijing, China; 20 Department of Infectious Disease, Peking University International Hospital, Beijing, China; 21 Humanity and Health Clinical Trial Center, Humanity and Health Medical Group, 9 Queen’s Road Central, Central, Hong Kong SAR, China; 22 Zhongshan Hospital, Fudan University, Shanghai, China

**Keywords:** HBsAg loss, HBsAg reduction, Chronic hepatitis B, Virological relapse, Adverse event, Confounding factor, HBV DNA, Barriers, Therapeutic endpoints, Immune restoration, cccDNA

## Abstract

**Background:**

Achieving a “functional” cure for chronic hepatitis B (HBV) is primary goal for novel antiviral treatments. We sought to evaluate efficacy and safety of these novel treatments and identified emerging barriers to achieving a functional cure.

**Approach:**

We systematically reviewed clinical trials from 2018 to 2023, identifying 244 trials from clinicaltrials.gov records on HBV. The primary outcome was functional cure rate at the end of follow-up (EOF). Secondary outcomes included changes in HBsAg levels, HBsAg loss rates, HBV DNA rebound, and adverse events. Meta-analysis was performed.

**Results:**

Our meta-analysis of 19 studies involving 1789 non-cirrhotic HBV patients found a minimal functional cure rate (0.0%, 95%CI 0.0–0.4%) and low HBsAg loss rates (0.9% at the end of treatment [EOT] and 0.1% at EOF). HBsAg levels declined at EOT (−0.41 log10 IU/mL, 95%CI −0.45 to −0.37, p < 0.001) but this reduction was not sustained to EOF. Virological relapse occurred in 20.5% of cases off-treatment. Although novel treatments were well-tolerated, they had higher adverse event rates (OR = 1.77, 95%CI 1.26–2.48). Challenges to achieving a functional cure include complex trial designs and unknown confounding factors.

**Conclusion:**

Novel antiviral treatments showed limited effectiveness in achieving HBsAg loss and reduction, highlighting the need to address identified barriers in future research.

**Supplementary Information:**

The online version contains supplementary material available at 10.1007/s12072-025-10823-5.

## Introduction

Chronic Hepatitis B (CHB) remains a significant global public health challenge, affecting 296 million individuals with 1.5 million new infections and 820,000 deaths in 2019, mainly due to cirrhosis and hepatocellular carcinoma (HCC) [1]. Despite a declining prevalence [2], hepatitis B virus (HBV)-related deaths are projected to rise to 17 million by 2030 [3].The World Health Organization (WHO) aims to eliminate viral hepatitis by 2030 through timely care linkage and universal vaccination [4], yet there is a lack of highly effective curative therapies for CHB.

In CHB patients, seroclearance of hepatitis B surface antigen (HBsAg) either spontaneous or after antiviral therapy, is of great importance as it allows sustained off-therapy disease remission with high durability (> 95% after greater than 5 years follow-up) [5]. To date, various treatment guidelines have accepted seroclearance of HBsAg as the most desirable intermediate treatment endpoint [6–8]. In keeping with this, the 2022 AASLD/EASL endpoints conference recommends the preferred primary endpoint for phase II/III trials evaluating finite treatments for CHB is a “functional” cure, defined as sustained HBsAg loss and HBV DNA less than the lower limit of quantitation (LLOQ) 24 weeks off-treatment [9]. Current HBV therapeutics with one year of pegylated-interferon-a (PEG-IFNa) and long-term nucleos(t)ide analogues (NAs), rarely achieve HBV cure [10]. Sustained functional cure after stopping NA therapy was observed in some Caucasian patients but rarely in Asian patients [11]. Switching from a NA to IFN therapy after HBV DNA suppression with NA increases the chance of HBsAg clearance mainly in those with low HBsAg levels [12].

The rapid evolution in our understanding of the HBV life cycle has accelerated the development of innovative anti-viral treatments for HBV infection [13]. However, it remains unclear if these novel anti-viral approaches lead to a higher rate of functional cure or HBsAg loss than existing registered therapies. Moreover, there is a pressing need to assess whether the ongoing drug development efforts align with the WHO goal of achieving HBV elimination and whether they represent a sound investment of resources. Therefore, this systematic review and meta-analysis seeks to assess the efficacy and safety of the novel anti-viral compounds and to identify barriers to “functional” cure.

## Methods

### Trial search

We conducted a thorough search of clinicaltrials.gov from January 1^st^, 2018, to December 1st 2023, identifying 244 records for interventional clinical trials on Hepatitis B Virus (HBV) across phases 1–3. After excluding trials that were not relevant, 90 records investigating 42 unique compounds remained (Supplementary p1).

We conducted a manual search for published full text articles in Pubmed and Google focusing on the 42 unique compounds. We excluded articles available only in abstract format. Additionally, we included novel compounds mentioned in review papers or grey literature if full articles were identified (Table S1). This review adhered to the Preferred Reporting Items for Systematic Reviews and Meta-Analyses (PRISMA) and the review was registered in PROSPERO (CRD42024529419) without a review protocol. We used only published data, so approval from an ethics committee was not required.

### Data extraction

Two independent reviewers (JC and GL) extracted data from each study and assessed data completeness. Any discrepancies were resolved through discussion or by involving an additional reviewer (JD). Extracted data included investigational compounds, trial phase, treatment regimens (dose and duration), patient numbers, and patient HBeAg status and treatment history. The primary outcome was rate of functional cure at the end of follow-up (EOF), calculated as the reported number of events divided by the sample size. The secondary outcomes included changes in quantification of HBsAg from baseline to the end of treatment (EOT) and EOF, rates of HBsAg loss at EOT and EOF, HBV DNA rebound during follow-up, and the number of adverse events (AE), serious adverse events (SAE) and deaths.

### Data standardization

To ensure consistency in outcomes reporting, continuous measurements such as means, and standard deviations were standardized across the studies. Standard deviations (SD) were converted into standard errors (SE) using the formula SE = SD/√(sample size) and vice versa. In cases where none of the SD and SE were reported for an individual study, a pooled standard deviation from all the other studies in our meta-analysis with available SD/SE was utilized, as recommended by Furukawa et al. [14].The detailed estimation is described in appendix p4. Binomial measurements were transformed into percentages by dividing the reported number of events by the sample size. The mean change in HBsAg from baseline to EOT and EOF was assumed to be zero when studies reported “no meaningful change from baseline”.

In studies comparing multiple treatment arms with varying doses to a single control arm, each treatment–control comparison was analysed separately. To avoid double-counting the sample size of the control arm, the sample size of the control arm was proportionately reduced for each comparison [15]. This approach maintains the integrity of the analysis by ensuring that each treatment dose is appropriately represented and compared against the placebo control group.

### Statistical analysis

We conducted the meta-analysis using Stata (version 18.0, StataCorp LLC). Binomial trial-level and arm-level data from treatment groups, including the rates of functional cure, HBsAg loss, HBV DNA rebound, and safety profiles (AE/SAE/Deaths), were pooled using STATA command "*metaprop*" [16]. This command computes 95% confidence intervals using the score statistic to prevent CIs from exceeding the 0–1 range. Moreover, it incorporates the Freeman-Tukey double arcsine transformation of proportions, allowing the inclusion of studies with rates equal to zero, which occurred frequently in our case.

We pooled trial-level and arm-level data in continuous format, specifically EOT and EOF decline in HBsAg level, using STATA command "*metan*" [17]. We used the DerSimonian–Laird (DL) model, which assumes that the true effect could vary across studies and individual-study effects are distributed with a variance of *τ*^2^ around an overall true effect.

To compare the differences between treatment and control arms, we utilized the STATA command “*meta esize*” [18]. We specified the effect size using the log odds ratio (STATA command “*lnorpeto*” defined by Peto et al.), which is preferable for rare events. The DerSimonian–Laird (DL) model was employed. In the case of continuous variables comparison, we utilized Hedges’s g standardized mean difference as recommended, applying a random-effects model [19]. To assess statistical heterogeneity, we utilized the I-square (I^2^) statistic [20]. In a situation that I-square equalled to zero, the random-effects model was reduced to fixed-effect model.

Meta-regression models were used to examine the association between baseline factors and outcomes as well as the dose-dependent response using Sidik–Jonkman method (Stata command “*meta regress*” [21]). Funnel-plot asymmetry (as an indication of potential publication bias) was assessed visually (using Funnel and Dio plot [22]) and statistically (using Egger's tests and LFK index).

## Results

### Trial characteristics

**Fig. 1:**
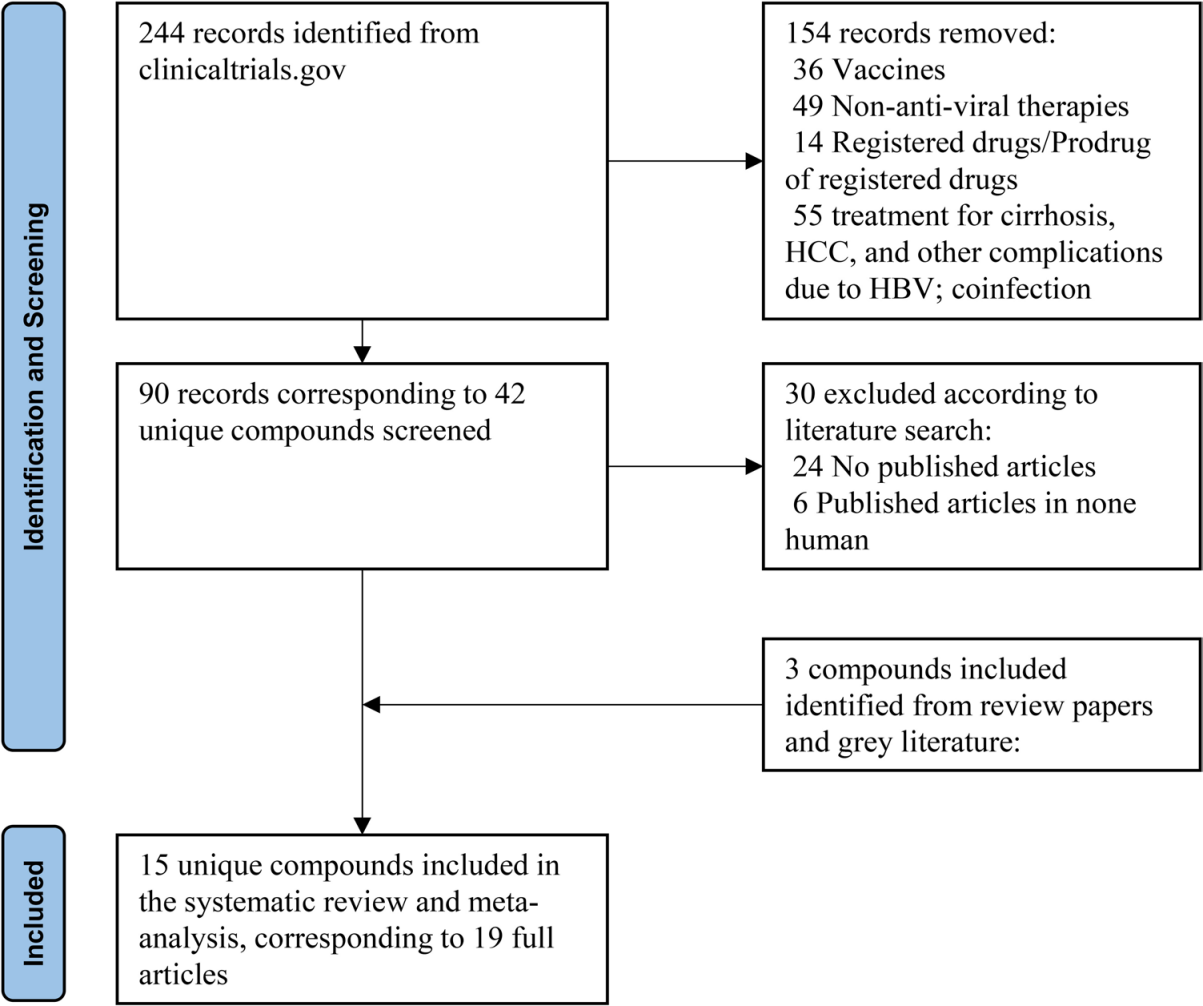
Flowchart of study selection

A total of 19 studies were identified, corresponding to 15 distinct compounds (Fig. 1). The characteristics of trials included in the meta-analysis are summarized in Table 1. Across all trials, participants exclusively comprised non-cirrhotic CHB patients, with 1505 individuals allocated to treatment arms and 284 to control arms. Baseline characteristics varied, with diverse HBeAg status (negative, positive, or both) and treatment history (treatment-naïve, treatment-experienced, or NAs-suppressed). Treatment protocols exhibited notable heterogeneity in duration (ranging from 28 days to 48 weeks), doses (ranging from 20 mg every 4 weeks to 800 mg once daily for 28 days), and administration methods (including injection and oral administration). Follow-up periods ranged from 4 to 48 weeks. Control arms received either placebo (study n = 9) or placebo in conjunction with NAs (study n = 10). The 19 studies represented 86 unique treatment arms.

**Table 1:** Characteristics of included trials

Author, year	Compounds	Regimens	Characteristics				qHBsAg (log10 IU/ml)			Loss of HBsAg (%)		Functional Cure	Rebound of HBV DNA	AE		
			Sample size	HBeAg	Tx history	Off-tx FU	BL	EOT change	EOF change	EOT	EOF			Any AE	SAE	Death
**Capsid assembly modulators (CAM)**																
Yuen MF 2022a [39]	Vebicorvir (ABI-H0731, Assembly Biosciences)	Vebicorvir 300 mg daily + NAs, 24 weeks	29	+	NA-suppressed	NR	3.48 (0.40)	0.028 (−0.009, 0.065)	NR	0 (0)	NR	NR	0 (0)	24 (53)	0 (0)	0 (0)
			16	−			2.99 (0.56)	0.087 (0.017, 0.156)	NR	0 (0)	NR	NR	0 (0)			
		Placebo + NAs, 24 weeks	18	+			3.57 (0.52)	0.041 (−0.008, 0.089)	NR	0 (0)	NR	NR	0 (0)	8 (29)	0 (0)	0 (0)
			10	−			3.35 (0.65)	0.009 (−0.080, 0.097)	NR	0 (0)	NR	NR	0 (0)			
Sulkowski MS 2022 [40]	Vebicorvir (ABI-H0731, Assembly Biosciences)	Vebicorvir 300 mg daily + ETV 0.5 mg, 24 weeks	13	+	Tx-naïve	NR	4.5 (0.5)	−0.169 (−0.359, 0.022)	NR	0 (0)	NR	NR	1 (7.7)	7 (54)	0 (0)	0 (0)
	Phase 2, terminated	Placebo + ETV, 24 weeks	12				4.7 (0.4)	− 0.220 (–0.419, –0.021)	NR	0 (0)	NR	NR	1 (8.3)	5 (42)	0 (0)	0 (0)
Zhang H 2021 [41]	Morphothiadin (GLS4, HEC Pharma)	GLS4 120 mg + Ritonavir 100 mg daily, 28 days	8	+	Tx-naïve	40 d	4.51 (0.67)	−0.06	NR	0 (0)	0 (0)	0 (0)	4 (50 (0))	5 (62.5)	0 (0)	0 (0)
		GLS4 240 mg + Ritonavir 100 mg daily, 28 days	8				4.21 (0.52)	-0.14	NR	0 (0)	0 (0)	0 (0)	5 (62.5)	6 (75)	2 (25)	0 (0)
	Phase 1b	ETV 0.5 mg daily [control]	8				3.77 (0.52)	−0.33	NR	0 (0)	0 (0)	0 (0)	2 (25)	7 (87.5)	0 (0)	0 (0)
Feld JJ 2022 [42]	EDP-514 (Enanta)	EDP-514 200 mg daily + NA, 28 d	6	Both	NA-suppressed	4 weeks	NR	No clinically meaningful changes	NR	NR	NR	NR	NA	5 (83.3)	0 (0)	NR
		EDP-514 400 mg daily + NA, 28 d	6				NR		NR	NR	NR	NR	NA	1 (16.7)	0 (0)	NR
		EDP-514 800 mg daily + NA, 28 d	6				NR		NR	NR	NR	NR	NA	2 (33.3)	1 (16.7)	NR
	Phase 1	Placebo + NAs	14				NR		NR	NR	NR	NR	NA	0	0 (0)	NR
Yuen MF 2024 [43]	EDP-514	EDP-514 QD, 200 mg	6	Both	Tx-naïve or stop IFN or NUC for > one year	8 wk	3.14 (3.12)	No clinically meaningful changes from baseline	NR	NR	NR	NR	0 (0)	2 (33.3)	0 (0)	0 (0)
		EDP-514 QD, 400 mg	6				4.38 (4.66)		NR	NR	NR	NR	0 (0)	3 (50)	0 (0)	0 (0)
		EDP-514 QD, 800 mg	7				3.84 (4.17)		NR	NR	NR	NR	0 (0)	2 (28.6)	0 (0)	0 (0)
	Phase 1	Placebo	6				3.42 (3.68)		NR	NR	NR	NR	0 (0)	2 (33.3)	0 (0)	0 (0)
Yuen MF 2022b [44]	AB-506 (Arbutus)	AB-506 160 mg QD, 28 d	10	Both	Tx-naïve or stop IFN/NA for > 6 mon	28 d	3.62 (0.56)	-0.021 (0.069)	NR	NR	NR	NR	NR	8 (80)	0 (0)	0 (0)
	Phase 1,	AB-506 400 mg QD, 28 d	10				4.23 (0.66)	0.113 (0.176)	NR	NR	NR	NR	NR	7 (70)	0 (0)	0 (0)
	terminated	Placebo	4				3.52 (0.60)	0.006 (0.07)	NR	NR	NR	NR	NR	3 (75)	0 (0)	0 (0)
Yuen MF 2021a [45]	RG7907, aka RO7049389 (Roche)	RO7049389 200 mg BD, 4 wk	6	Both	Tx-naïve or stop IFN/NA for > 6 mon	48 weeks	3.61 (0.72)	−0.04 (0.07)	NR	NR	NR	NR	100%	4 (67)	0 (0)	0 (0)
		RO7049389 400 mg BD, 4 wk	6				4.31 (0.55)	0.02 (0.12)	NR	NR	NR	NR	100%	4 (67)	0 (0)	0 (0)
		RO7049389 200 mg QD, 4 wk	6				3.69 (1.51)	0.01 (0.07)	NR	NR	NR	NR	100%	2 (33)	0 (0)	0 (0)
	Phase 1, Phase 2 dropped by Roche	RO7049389 600 mg QD, 4 wk	6				3.47 (1.17)	0.04 (0.08)	NR	NR	NR	NR	100%	5 (83)	0 (0)	0 (0)
		RO7049389 1000 mg QD, 4 wk	7				3.16 (0.98)	0.06 (0.08)	NR	NR	NR	NR	100%	4 (57)	0 (0)	0 (0)
		Placebo	6				3.38 (0.75)	−0.02 (0.07)	NR	NR	NR	NR	0 (0)	3 (50)	0 (0)	0 (0)
Agarwal K 2023 [46]	ABI-H2158, Assembly Biosciences	ABI-H2158 100 mg QD, 14 d	7	+	Tx-naïve	6 week	4.2 (2.8, 5.4)	0.03 (0.054)	NR	NR	NR	NR	NR	3 (42.9)	0 (0)	0 (0)
		ABI-H2158 300 mg QD, 14 d	7				4.8 (4.4, 5.0)	0.01 (0.045)	NR	NR	NR	NR	NR	2 (28.6)	0 (0)	0 (0)
		ABI-H2158 500 mg QD, 14 d	7				4.8 (4.3, 5.4)	−0.04 (0.069)	NR	NR	NR	NR	NR	4 (57.1)	1 (14.3)	0 (0)
	Phase 1a/b, terminated	ABI-H2158 300 mg BID, 14 d	8				4.5 (3.6, 5.0)	0.00 (0.038)	NR	NR	NR	NR	NR	4 (50.0)	1 (12.5)	0 (0)
		Placebo	8				4.7 (4.1, 5.1)	–0.04 (0.058)	NR	NR	NR	NR	NR	3 (37.5)	0 (0)	0 (0)
Jia H 2023 [47]	ZMH1505R	ZMH1505R, 50 mg	8	Both	Tx-naïve or stop IFN/NA for > 12/6 mon	28 days	4.23 (0.62)	No decrease	NR	NR	NR	NR	NR	3 (37.5)	0 (0)	NR
	(Canocapavir)	ZMH1505R, 100 mg	8				4.10 (1.00)		NR	NR	NR	NR	NR	5 (62.5)	0 (0)	NR
	Phase 1b	ZMH1505R, 200 mg	8				3.97 (0.75)		NR	NR	NR	NR	NR	4 (50)	0 (0)	NR
		Placebo	6				3.79 (1.17)		NR	NR	NR	NR	NR	3 (50)	0 (0)	NR
**siRNA agents**																
Yuen MF 2023 [23]	JNJ-56136379 (JNJ-6379, JNJ-3989, Janssen, REEF-1)	NA/JNJ-6379 250 mg QD, 48 wk	8	+	Tx-naïve	24 weeks	4·19 (0·29)	–0·43 (0·31)	–0·40 (0·29)	0 (0)	0 (0)		27 overall	41 (85)	0 (0)	0 (0)
		NA/JNJ-6379 250 mg QD, 48 wk	10	−	Tx-naïve		3·81 (0·19)	–0·03 (0·07)	–0·15 (0·07)	0 (0)	0 (0)					
		NA/JNJ-6379 250 mg QD, 48 wk	7	+	NA-suppressed		3·23 (0·11)	0·02 (0·03)	–0·04 (0·03)	0 (0)	0 (0)					
		NA/JNJ-6379 250 mg QD, 48 wk	23	−	NA-suppressed		3·49 (0·13)	0·01 (0·08)	–0·09 (0·02)	0 (0)	0 (0)					
		NA/JNJ-3989 40 mg injection Q4W, 48 wk	15	+	Tx-naïve		4·61 (0·15)	–1·77 (0·14)	–1·32 (0·20)	0 (0)	0 (0)			69 (74)	0 (0)	0 (0)
		NA/JNJ-3989 40 mg injection Q4W, 48 wk	18	−	Tx-naïve		3·95 (0·10)	–1·40 (0·13)	–0·90 (0·11)	0 (0)	0 (0)					
		NA/JNJ-3989 40 mg injection Q4W, 48 wk	15	+	NA-suppressed		3·88 (0·09)	–1·27 (0·11)	–0·91 (0·09)	0 (0)	0 (0)					
		NA/JNJ-3989 40 mg injection Q4W, 48 wk	43	−	NA-suppressed		3·52 (0·09)	–1·51 (0·07)	–0·98 (0·06)	0 (0)	0 (0)					
		NA/JNJ-3989 100 mg injection Q4W, 48wk	14	+	Tx-naïve		4·19 (0·33)	–2·55 (0·26)	–2·10 (0·37)	0 (0)	1/13 (7·7)			66 (71)	0 (0)	0 (0)
		NA/JNJ-3989 100 mg injection Q4W, 48wk	19	−	Tx-naïve		3·88 (0·16)	–2·16 (0·16)	–1·53 (0·19)	1/18 (5·6)	2/15 (13·3)					
		NA/JNJ-3989 100 mg injection Q4W, 48wk	11	+	NA-suppressed		3·50 (0·19)	–2·01 (0·17)	–1·39 (0·18)	0 (0)	0 (0)					
		NA/JNJ-3989 100 mg injection Q4W, 48wk	48	−	NA-suppressed		3·52 (0·09)	–1·93 (0·07)	–1·34 (0·08)	0 (0)	0 (0)					
		NA/JNJ-3989 200 mg injection Q4W, 48 wk	16	+	Tx-naïve		4·66 (0·13)	–3·56 (0·35)	–2·63 (0·34)	2/14 (14·3)	0 (0)	1/91 (1.0)		62 (65)	1 (1)	0 (0)
		NA/JNJ-3989 200 mg injection Q4W, 48 wk	19	−	Tx-naïve		4·04 (0·05)	–2·22 (0·14)	–1·48 (0·14)	0 (0)	0 (0)					
		NA/JNJ-3989 200 mg injection Q4W, 48 wk	14	+	NA-suppressed		3·74 (0·19)	–2·61 (0·18)	–2·18 (0·26)	0 (0)	1/14 (7·1)					
		NA/JNJ-3989 200 mg injection Q4W, 48 wk	45	−	NA-suppressed		3·47 (0·10)	–2·41 (0·14)	–1·62 (0·11)	1/45 (2·2)	0 (0)					
	Phase 2b	NA/JNJ-3989 100 mg Q4W/JNJ-6379 250 mg QD, 48 wk	13	+	Tx-naïve		4·63 (0·10)	–2·52 (0·27)	–2·04 (0·26)	0 (0)	0 (0)			68 (72)	1 (1)	0 (0)
		NA/JNJ-3989 100 mg Q4W/JNJ-6379 250 mg QD, 48 wk	20	−	Tx-naïve		4·02 (0·10)	–1·40 (0·11)	–1·08 (0·11)	0 (0)	0 (0)					
		NA/JNJ-3989 100 mg Q4W/JNJ-6379 250 mg QD, 48 wk	14	+	NA-suppressed		3·46 (0·13)	–1·78 (0·13)	–1·43 (0·14)	0 (0)	0 (0)					
		NA/JNJ-3989 100 mg Q4W/JNJ-6379 250 mg QD, 48 wk	47	−	NA-suppressed		3·29 (0·08)	–1·71 (0·08)	–1·30 (0·08)	0 (0)	0 (0)					
		Placebo + NA	7	+	Tx-naïve		4·37 (0·40)	–0·80 (0·80)	–1·02 (0·98)	0 (0)	1/7 (14·3)	1/45 (2.2)		30 (67)	0 (0)	0 (0)
		Placebo + NA	9	−	Tx-naïve		4·04 (0·14)	–0·13 (0·11)	–0·13 (0·11)	0 (0)	0 (0)					
		Placebo + NA	6	+	NA-suppressed		3·59 (0·33)	–0·08 (0·03)	–0·14 (0·05)	0 (0)	0 (0)					
		Placebo + NA	23	−	NA-suppressed		3·64 (0·12)	–0·11 (0·03)	–0·11 (0·03)	0 (0)	0 (0)					
Janssen H 2023 [48]	JNJ-56136379 (JNJ-6379) (JADE) (Janssen)	JNJ-56136379 75 mg QD, 24 wk	28	Both	Tx-naïve	48 weeks	4.00 (0.71)	No relevant effect	No relevant effect	0 (0)	0 (0)	0 (0)	6/28 (21.4)	18 (64)	0 (0)	NR
		JNJ-56136379 75 mg QD + NA, 24 wk	33		Tx-naïve		3.97 (0.69)	HBeAg+:−0.14 (0.10) HBeAg-: 0.04 (0.02)	HBeAg+: 0.06 (0.48) HBeAg−:−0.02 (0.03)	0 (0)	0 (0)	0 (0)	1/66 (1.5)	55 (83)	3 (5)	NR
		JNJ-56136379 75 mg QD + NA, 24 wk	33		NA-suppressed		3.48 (0.61)	HBeAg +: −0.06 (0.08) HBeAg−: −0.02 (0.01)	HBeAg+: −0.08 (0.09) HBeAg−: −0.04 (0.02)	0 (0)	0 (0)	0 (0)				NR
		JNJ-56136379 250 mg QD, 24 wk	32		Tx-naïve		4.13 (0.53)	No relevant effect	HBeAg+: −0.04 (0.27) HBeAg−: 0.01 (0.03)	0 (0)	0 (0)	0 (0)	1/32 (3.1)	25 (78)	0 (0)	NR
		JNJ-56136379 250 mg QD + NA, 24 wk	33		Tx-naïve		3.98 (0.66)	HBeAg + : −0.41 (0.15) HBeAg-: 0.09 (0.04)	HBeAg+: −0.81 (0.45) HBeAg−: 0.08 (0.04)	0 (0)	0 (0)	0 (0)	5/95 (5.3)	54 (86)	4 (6)	NR
	Phase 2	JNJ-56136379 250 mg QD + NA, 24 wk	30		NA-suppressed		3.49 (0.53)	HBeAg+: 0.11 (0.06) HBeAg−: 0.09 (0.01)	HBeAg+: 0.05 (0.07) HBeAg−: 0.03 (0.02)	0 (0)	0 (0)	0 (0)				NR
		Placebo + NA	22		Tx-naïve		4.05 (0.65)	HBeAg+: − 0.25 (0.11) HBeAg-: 0.02 (0.02)	HBeAg+: −0.11 (0.15) HBeAg−: 0.03 (0.03)	0 (0)	0 (0)	0 (0)	1/43 (2.3)	34 (79)	1 (2)	NR
		Placebo + NA	21		NA-suppressed		3.58 (0.44)	HBeAg+: 0.01 (0.06) HBeAg-: 0.02 (0.02)	HBeAg+: 0.04 (0.06) HBeAg−: 0.01 (0.02)	0 (0)	0 (0)	0 (0)				NR
Yuen MF 2022c [49]	JNJ-73763989 (JNJ-3989) ± JNJ-56136379 (JNJ-6379) (Janssen)	JNJ-3989 25 mg Q4W + NA QD, 12–16 wk	8	Both	Both	40–44 weeks	3.13 (1.26)	–1.0 (0.2)	–0.6 (0.1)	NR	NR	NR	NR	6 (75.0)	0 (0)	0 (0)
		JNJ-3989 50 mg Q4W + NA QD, 12–16 wk	8				3.33 (0.66)	–1.2 (0.1)	–0.7 (0.1)	NR	NR	NR	NR	4 (50.0)	0 (0)	0 (0)
		JNJ-3989 100 mg Q4W + NA QD, 12–16 wk	8				2.93 (0.96)	–1.5 (0.2)	–0.8 (0.2)	NR	NR	NR	NR	4 (50.0)	1/8 (12.5)	0 (0)
		JNJ-3989 200 mg Q4W + NA QD, 12–16 wk	8				2.50 (1.32)	–1.8 (0.2)	–1.0 (0.2)	NR	NR	NR	NR	3 (37.5)	0 (0)	0 (0)
		JNJ-3989 300 mg Q4W + NA QD, 12–16 wk	8				3.04 (0.85)	–1.5 (0.1)	–0.7 (0.1)	NR	NR	NR	NR	6 (75.0)	0 (0)	0 (0)
		JNJ-3989 400 mg Q4W + NA QD, 12–16 wk	8				3.18 (0.80)	–1.8 (0.2)	–0.9 (0.1)	NR	NR	NR	NR	7 (87.5)	2/8 (25)	0 (0)
		JNJ-3989 300 mg Q4W + NA QD, 12–16 wk	4	+	Tx-naïve		4.81 (0.65)	–2.2 (0.5)	–2.1 (0.7)	NR	NR	NR	NR	2 (50.0)	0 (0)	0 (0)
		JNJ-3989 300 mg Q4W + NA QD, 12–16 wk	4	+	NA-suppressed		3.76 (0.37)	–2.4 (0.3)	–2.3 (0.6)	NR	NR	NR	NR	2 (50.0)	0 (0)	0 (0)
		JNJ-3989 200 mg Q4W + NA QD + JNJ-6379 250 mg QD, 12 wk	12	Both	Both		3.04 (0.79)	–1.7 (0.1)	–1.2 (0.2)	NR	NR	NR	NR	2 (16.7)	0 (0)	0 (0)
		JNJ-3989 100 mg Q2W + NA QD, 12–16 wk	4				2.72 (0.60)	–1.5 (0.2)	–0.9 (0.2)	NR	NR	NR	NR	2 (50.0)	0 (0)	0 (0)
		JNJ-3989 100 mg QW + NA QD, 12–16 wk	4				3.15 (0.24)	–1.2 (0.3)	–0.4 (0.2)	NR	NR	NR	NR	4 (100.0)	0 (0)	0 (0)
	Phase 2a, JNJ-3989 was dropped by JnJ	JNJ-3989 200 mg QW + NA QD, 12–16 wk	4				3.00 (1.07)	–2.1 (0.3)	–1.5 (0.1)	NR	NR	NR	NR	3 (75.0)	0 (0)	0 (0)
		JNJ-3989 300 mg QW + NA QD, 12–16 wk	4				3.24 (0.68)	–1.9 (0.1)	–1.1 (0.3)	NR	NR	NR	NR	2 (50.0)	0 (0)	0 (0)
Gane E 2023a [50]	VIR -2218 (Vir Biotechnology)	VIR-2218 Q4W 20 mg + NA, 4 wk	3	-	Tx-experienced	12 weeks	3.3 (0.3)	NR	NR	0 (0)	0 (0)	0 (0)	NR	0 (0)	0 (0)	0 (0)
		VIR-2218 Q4W 50 mg + NA, 4 wk	6	-			3.3 (0.5)	NR	NR	0 (0)	0 (0)	0 (0)	NR	2 (33)	0 (0)	0 (0)
		VIR-2218 Q4W 50 mg + NA, 4 wk	3	+			3.5 (0.3)	NR	NR	0 (0)	0 (0)	0 (0)	NR	2 (67)	0 (0)	0 (0)
		VIR-2218 Q4W 100 mg + NA, 4 wk	6	-			3.4 (0.5)	NR	-0.75 (0.51)	0 (0)	0 (0)	0 (0)	NR	5 (83)	1 (17)	0 (0)
		VIR-2218 Q4W 200 mg + NA, 4 wk	3	-			3.9 (0.6)	NR	-0.87 (0.55)	0 (0)	0 (0)	0 (0)	NR	2 (67)	0 (0)	0 (0)
		VIR-2218 Q4W 200 mg + NA, 4 wk	3	+			3.3 (0.4)	NR	NR	0 (0)	0 (0)	0 (0)	NR	2 (67)	0 (0)	0 (0)
	Phase 1/2	Placebo + NA	6	-			3.5 (0.4)	NR	NR	0 (0)	0 (0)	0 (0)	NR	1 (17)	0 (0)	0 (0)
		Placebo + NA	2	+			3.2 (0.3)	NR	NR	0 (0)	0 (0)	0 (0)	NR	1 (50)	0 (0)	0 (0)
Gane E 2023b [51]	RG6346, aka RO7445482 (Roche/Dicerna)	Single dose (3 mg/kg), 12wk	6	Both	Tx naïve	12 mon	4.16 (0.43)	-0.69		0 (0)	0 (0)	0 (0)	NR	5 (83.3)	0 (0)	NR
		Placebo	3				3.82 (0.86)	0		0 (0)	0 (0)	0 (0)	NR	1 (33.3)	0 (0)	NR
		4 × QM doses (1.5 mg/kg), 16wk	4		NA-suppressed		3.48 (0.29)	-1.39	3/12 (25.0%)	0 (0)	0 (0)	0 (0)	NR	2 (50.0)	0 (0)	NR
		4 × QM doses (3 mg/kg), 16wk	4				3.62 (0.59)	-1.8		0 (0)	0 (0)	0 (0)	NR	4 (100)	0 (0)	NR
	Phase 1, move to Phase 2	4 × QM doses (6 mg/kg), 16wk	4				3.45 (0.31)	-1.64		0 (0)	0 (0)	0 (0)	NR	1 (25.0)	0 (0)	NR
		Placebo	6				3.71 (0.26)	-0.01		0 (0)	0 (0)	0 (0)	NR	5 (83.3)	0 (0)	NR
**Antisense oligonucleotides**																
Yuen MF 2021b [25]	Bepirovirsen (GSK3228836, GSK)	Bepirovirsen 150 mg Q2W for first 2 weeks & QW for the rest 2 weeks + NA post-Bepirovirsen, 28 d	6	Both	Tx naïve	26 wk	3.57 (1.244)	–0.50 (0.57)	NR	0 (0)	0 (0)	0 (0)	0 (0)	5 (83.3)	0 (0)	NR
		Bepirovirsen 300 mg Q2W for first 2 weeks & QW for the rest 2 weeks + NA post-Bepirovirsen, 28d	12				3.89 (1.056)	–1.56 (1.38)	NR	2/12 (16.7)	0 (0)	0 (0)	0 (0)	6 (50.0)	1/12 (8.3)	NR
		Placebo	6				3.21 (1.30)	0.00 (0.11)	NR	0 (0)	0 (0)	0 (0)	0 (0)	3 (50.0)	0 (0)	NR
	Phase 2a	Bepirovirsen 300 mg QW + NA, 28d	5		Tx-experience		2.78 (0.363)	–1.99 (1.8)	NR	2/5 (40 (0).0 (0))	0 (0)	0 (0)	0 (0)	3 (60.0)	0 (0)	NR
		Placebo + NA	2				3.77 (1.02)	–0.01 (0.04)	NR	0 (0)	0 (0)	0 (0)	0 (0)	1 (50.0)	0 (0)	NR
Yuen MF 2022d [24]	Bepirovirsen (GSK3228836, GSK) B-clear	Bepirovirsen 300 mg QW + NA, 24 wk	68	Both	Tx-experience	24 weeks	3.29 (0.62)	HBsAg < LLQD 17 (26)	HBsAg < LLQD 8 (12)	HBsAg < LLQD 17 (26)	HBsAg < LLQD 8 (12)	6 (9)	NR	56 (82)	1 (1)	0 (0)
		Bepirovirsen 300 mg QW, 24 wk	70		Tx naïve		3.72 (0.77)	20 (29)	9 (14)	20 (29)	9 (14)	7 (10)	NR	65 (93)	6 (9)	0 (0)
		Bepirovirsen 300 mg QW 12wk + 150 mg QW 12wk + NA	68		Tx-experience		3.26 (0.61)	11 (16)	6 (9)	11 (16)	6 (9)	6 (9)	NR	59 (88)	1 (1)	0 (0)
		Bepirovirsen 300 mg QW 12wk + 150 mg QW 12wk	68		Tx naïve		3.65 (0.72)	8 (12)	4 (6)	8 (12)	4 (6)	4 (6)	NR	60 (90)	2 (3)	0 (0)
		Bepirovirsen 300 mg QW 12wk + placebo + NA	68		Tx-experience		3.33 (0.59)	4 (6)	3 (4)	7 (10)	3 (4)	2 (3)	NR	53 (78)	4 (6)	0 (0)
	Phase 2b, to Phase 3	Bepirovirsen 300 mg QW 12wk + placebo	68		Tx naïve		3.66 (0.67)	3 (4)	1(1)	7 (10)	1(1)	1 (1)	NR	62 (91)	3 (4)	0 (0)
		Placebo + NA	23		Tx-experience		3.43 (0.43)	4 (17)	0	4 (17)	0 (0)	0 (0)	NR	16 (70)	0 (0)	0 (0)
		Placebo	24		Tx naïve		3.76 (0.79)	5 (21)	1 (4)	5 (21)	1 (4)	0 (0)	NR	19 (79)	0 (0)	0 (0)
Yuen MF 2022e [52]	GSK3389404	30 mg Q1W	6	Both	Tx-naïve, Tx-experience, on-NUC	24 week	2.73 (0.68)	–0.1312 (0.07)	–0.0295 (0.04)	0 (0)	0 (0)	NR	NR	4 (67)	0 (0)	0 (0)
		60 mg Q1W	20				2.96 (0.54)	–0.3374 (0.32)	–0.0975 (0.08)	0 (0)	0 (0)	NR	NR	18 (90)	0 (0)	0 (0)
	Phase 2a	120 mg Q1W	15				2.82 (0.49)	–0.7531 (0.65)	–0.1822 (0.22)	0 (0)	0 (0)	NR	NR	12 (80)	1 (7)	0 (0)
		120 mg Q2W	15				3.00 (0.49)	–0.4429 (0.50)	–0.1038 (0.23)	0 (0)	0 (0)	NR	NR	11 (73)	2 (13)	0 (0)
		Placebo	10				3.15 (0.75)	–0.0212 (0.08)	–0.0046 (0.09)	0 (0)	0 (0)	NR	NR	9 (90)	1 (10)	0 (0)
Gane E 2021 [53]	RO7062931 (Roche)	0.5 mg/kg QM × 2 doses	6	Both	Tx-experience	16 weeks	3.4 (0.36)	NR	Almost all went back to the baseline	NR	NR	NR	NR	2 (33.3)	0 (0)	0 (0)
		1.5 mg/kg QM × 2 doses	7				3.4 (0.29)	NR		NR	NR	NR	NR	6 (85.7)	0 (0)	0 (0)
		3.0 mg/kg QM × 2 doses	6				3.4 (0.13)	− 0.28 (0.099)		NR	NR	NR	NR	4 (66.7)	0 (0)	0 (0)
		3.0 mg/kg QW × 5 doses	14				3.7 (0.36)	− 0.50 (0.209)		NR	NR	NR	NR	10 (71.4)	0 (0)	0 (0)
		3.0 mg/kg Q2W × 3 doses	7				3.7 (0.38)	− 0.39 (0.159)		NR	NR	NR	NR	4 (57.1)	0 (0)	0 (0)
	Phase 1	4.0 mg/kg QW × 4 doses	4				3.7 (0.27)	−0.34		NR	NR	NR	NR	3 (75.0)	0 (0)	0 (0)
		Placebo	15				3.6 (0.42)	−0.10 (0.071)		NR	NR	NR	NR	8 (53.3)	0 (0)	0 (0)
**ASPINs**																
Squires KE 2022 [54]	ATI-2173 (Antios)	ATI-2173 10 mg, QD, 28d	6	Both	Tx naïve	24 weeks	3.53 (0.48)	−0.11 (0.40)	NR	NR	NR	NR	-	1 (17)	NR	NR
		ATI-2173 25 mg, QD, 28d	5				3.37 (0.36)	−0.07 (0.12)	NR	NR	NR	NR	1 (25)	3 (60)	NR	NR
		ATI-2173 50 mg, QD, 28d	6				3.90 (0.69)	−0.09 (0.10)	NR	NR	NR	NR	0 (0)	4 (67)	NR	NR
	Phase 1b	Placebo	7				3.87 (0.84)	−0.08 (0.11)	NR	NR	NR	NR	NR	5 (71)	NR	NR

Data for qHBsAg are presented as mean (SD) or mean (95% CI); data for rates are presented as n (%)

*NR*: note reported

### Rate of functional cure

**Fig. 2:**
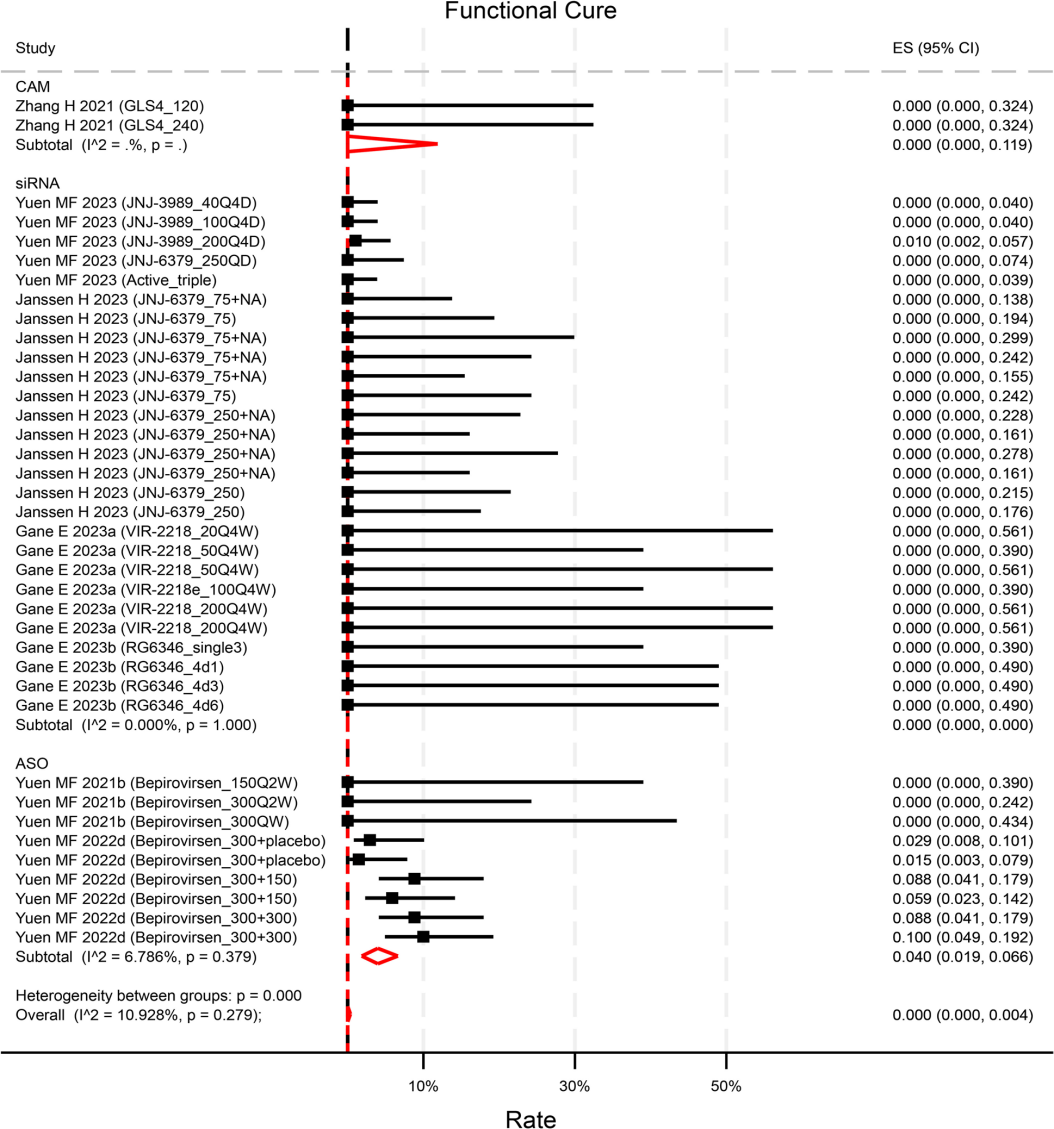
Reported rate of functional cure by compounds categories. Though numerically higher, the rate in ASO was not statistically significant than that in CAM (p = 0.81) and in siRNA (p = 0.35). p values were estimated by meta regression

Seven studies explicitly described the rate of functional cure at the end of follow-up (EOF). Among them, only 2 studies reported a non-zero functional cure rate. One small interfering RNA (siRNA) compound demonstrated a 1.0% (95%CI 0.2 5.7%) rate solely in the highest dose group [23]. Conversely, one antisense oligonucleotides (ASO) exhibited an overall rate of 6.3% (95%CI 4.4–9.1%) at EOF, with rates ranging from 1.5% (95%CI 0.3–7.9%) in treatment-naïve patients with the lowest dose to 10.0% (95%CI 4.9–19.2%) in treatment-experienced patients with the highest dose[24] (Fig. 2).A pooled rate of 0.0% (95%CI 0.0–0.4%, p = 0.99, I^2^ = 10.9%) of functional cure was observed across 7 studies with reported rates (contributing to 38 unique treatment arms of varying test doses) (Fig. 2). The rates did not differ between agents or baseline HBsAg level (Figure S1). When compared to NA-treated control groups, the novel antiviral treatments (contributing to 34 unique treatment arms) did not significantly enhance the rate of functional cure, yielding an odds ratio (OR) of 1.35 (95%CI 0.59–3.06, p = 0.48) (Figure S2).

### Rate of HBsAg loss and level of HBsAg decline

**Fig. 3:**
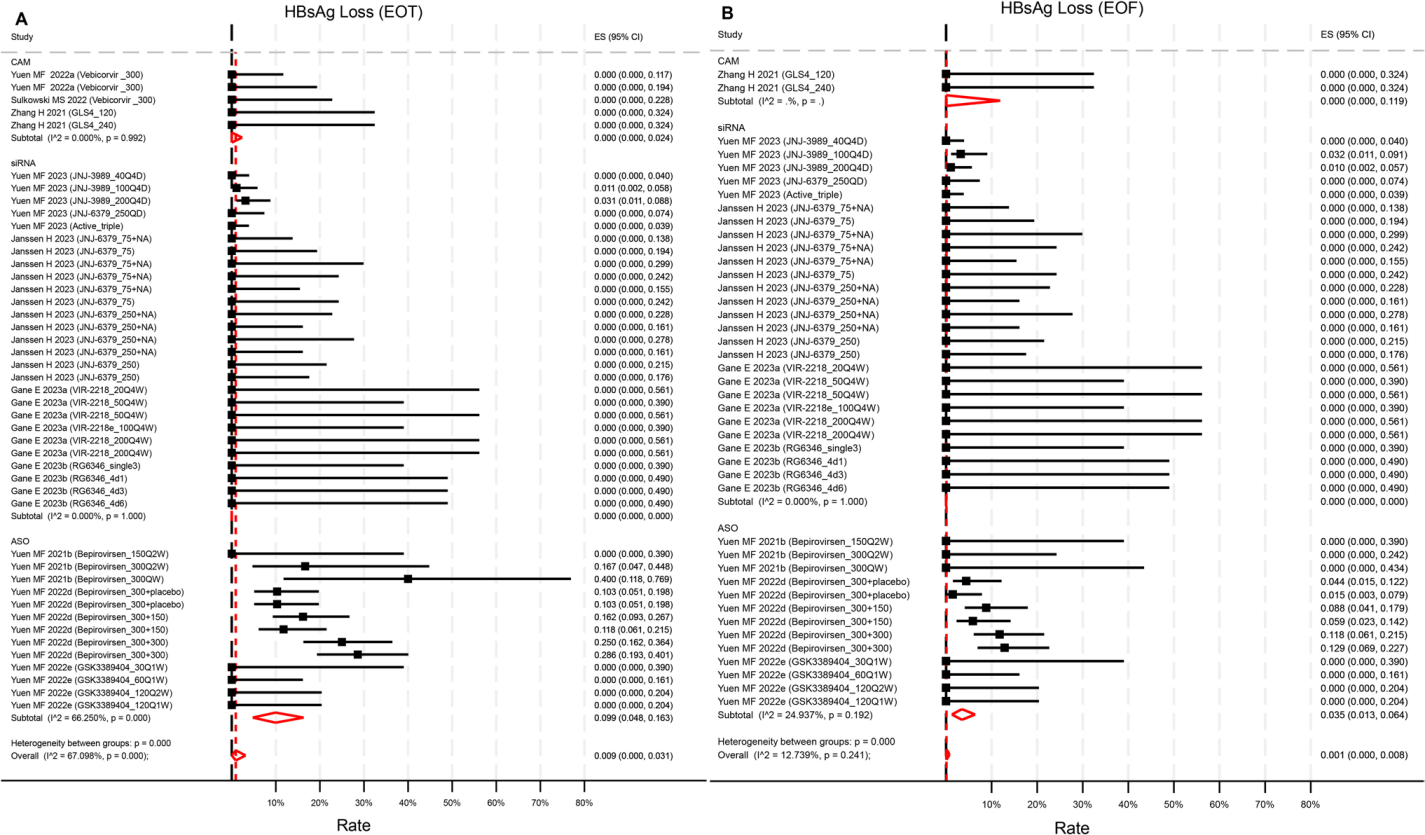
Reported rate of HBsAg loss **A** at end of treatment, **B** at end of follow up by compounds categories. The rate at EOT in ASO was statistically significantly higher than that in siRNA (p = 0.019) but not than that in CAM (p = 0.24). The rate at EOF was not statistically significantly different across categories. p values were estimated by meta regression

Ten studies reported HBsAg loss rates at EOT, with 8 also providing EOF rates. However, only one siRNA trial [23] and two ASO trials [24, 25] reported non-zero HBsAg loss rates at EOT. Pooled HBsAg loss at EOT was 0.9% (95%CI 0.0–3.1%, p = 0.064, I^2^ = 67.1%) across 10 studies with 45 unique treatment arms (Fig. 3a). Meanwhile, pooled HBsAg loss at EOF was 0.1% (95%CI 0–0.8%, p = 0.36, I^2^ = 12.7%) across 8 studies with 42 unique treatment arms (Fig. 3b). ASO compounds achieved a significantly higher rate of HBsAg loss at EOT compared to siRNA (p = 0.019), but not at EOF. Baseline HBsAg level did not influence the rates of HBsAg loss at EOT and EOF (Figure S3). Novel treatments did not significantly improve HBsAg loss compared to NA-treated control groups at either EOT (OR = 1.02, 95%CI 0.57–1.81, p = 0.94) or EOF (OR = 1.26, 95%CI 0.57–2.78, p = 0.56) (Figure S4).

**Fig. 4:**
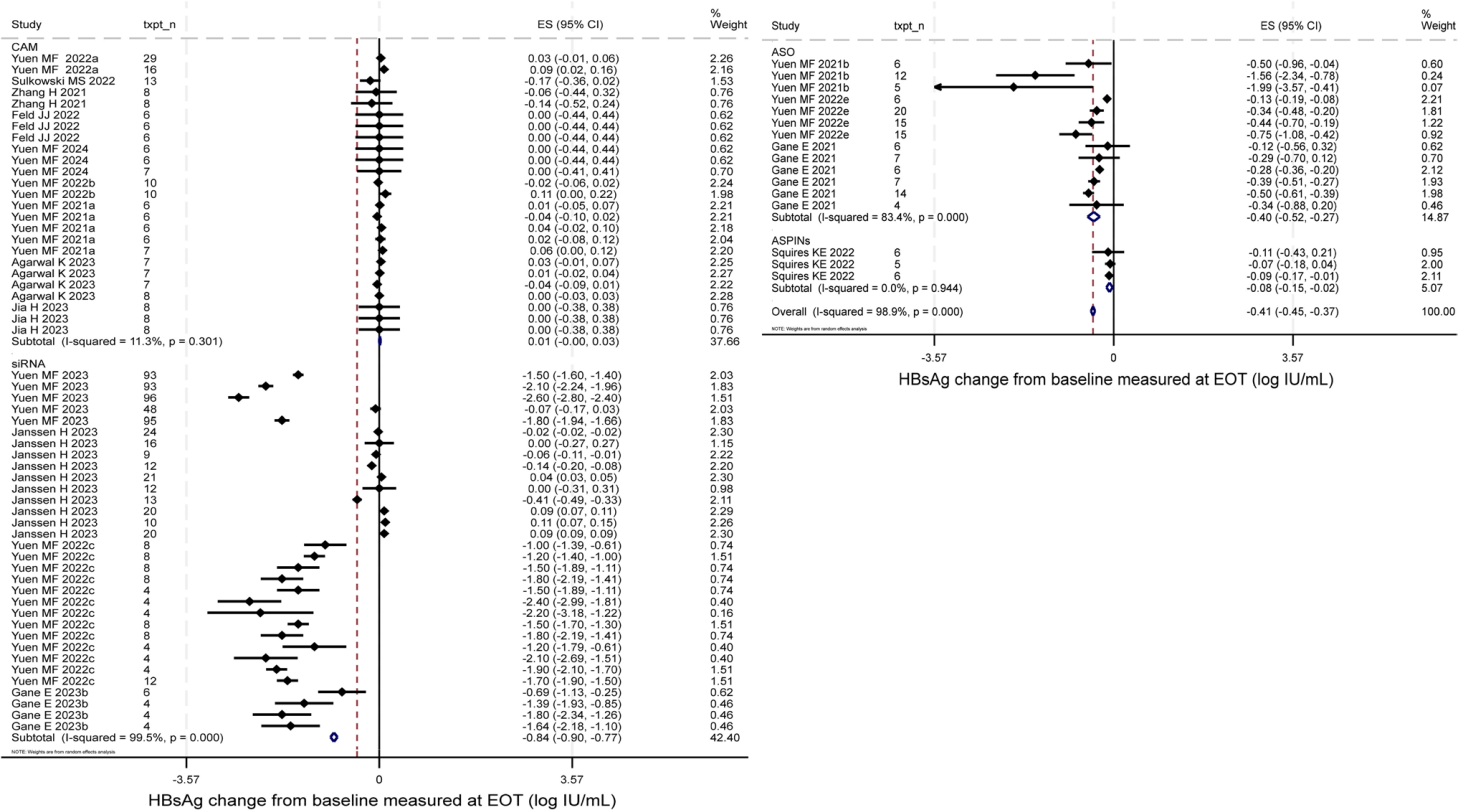
HBsAg change from baseline measured at EOT by compounds categories. siRNA induced a significantly larger reduction compared to ASO (p = 0.007), CAM (p < 0.001) and ASPINs (p = 0.009). p value was estimated by meta regression

Seventeen of the 19 studies reported HBsAg change from baseline to EOT, while six reported HBsAg change from baseline to EOF. Among the 17 studies with reported EOT HBsAg change, 10 studies (59%) reported a decline (negative change) while the other 7 reported an increase (positive change) or no change. Average EOT HBsAg change across the 17 studies (73 unique treatment arms) was −0.41 log10 IU/mL (95%CI −0.45 to −0.37, p < 0.001) with significant between-study heterogeneity (I^2^ = 98.9%, p < 0.001) (Fig. 4). siRNA compounds achieved a significantly larger reduction in EOT HBsAg change. Low baseline HBsAg level favors EOT HBsAg decline (Figure S5). Compare to NA-treated control groups (7 studies contributing to 26 unique treatment arms), novel treatments did not induce a significantly greater decline in HBsAg EOT (Figure S6). Nonetheless, when comparing to placebo control groups (9 studies contributing to 34 unique treatment arms), novel anti-viral treatments significantly induced a larger decline in HBsAg EOT (SMD = −0.35, 95%CI −0.63 to −0.06, p = 0.02, I^2^ = 14.4%) (Figure S6).

Only looking at studies reporting a decline, average EOT HBsAg decline across the 10 studies (contributing to 48 unique treatment arms of varying test doses) was −0.85 log10 IU/mL (95%CI −0.98 to −0.72, p < 0.001) (Figure S7). However, the decline was not sustained at EOF. Among studies with both reported negative EOT and EOF change from baseline (five studies, contributing to 26 unique treatment arms), the pooled EOT decline from baseline was -1.20 log10 IU/mL (95%CI −1.44 to −0.96, p < 0.001, I^2^ = 99.5%) while it was -0.64 log10 IU/mL (95%CI −0.73 to -0.56, p < 0.001, I^2^ = 98.6%) EOF, indicating a rebound in HBsAg levels post-treatment (Figure S8).

Meta-regression revealed a reverse dose–response effect (meta-regression coefficient = 0.0000103, p = 0.002, tau^2^ = 0.47, Adj R-squared = 12.0%) on HBsAg decline where the higher the dose the smaller the decline, though the effect is very tiny (Figure S9).

### Safety profile

**Fig. 5:**
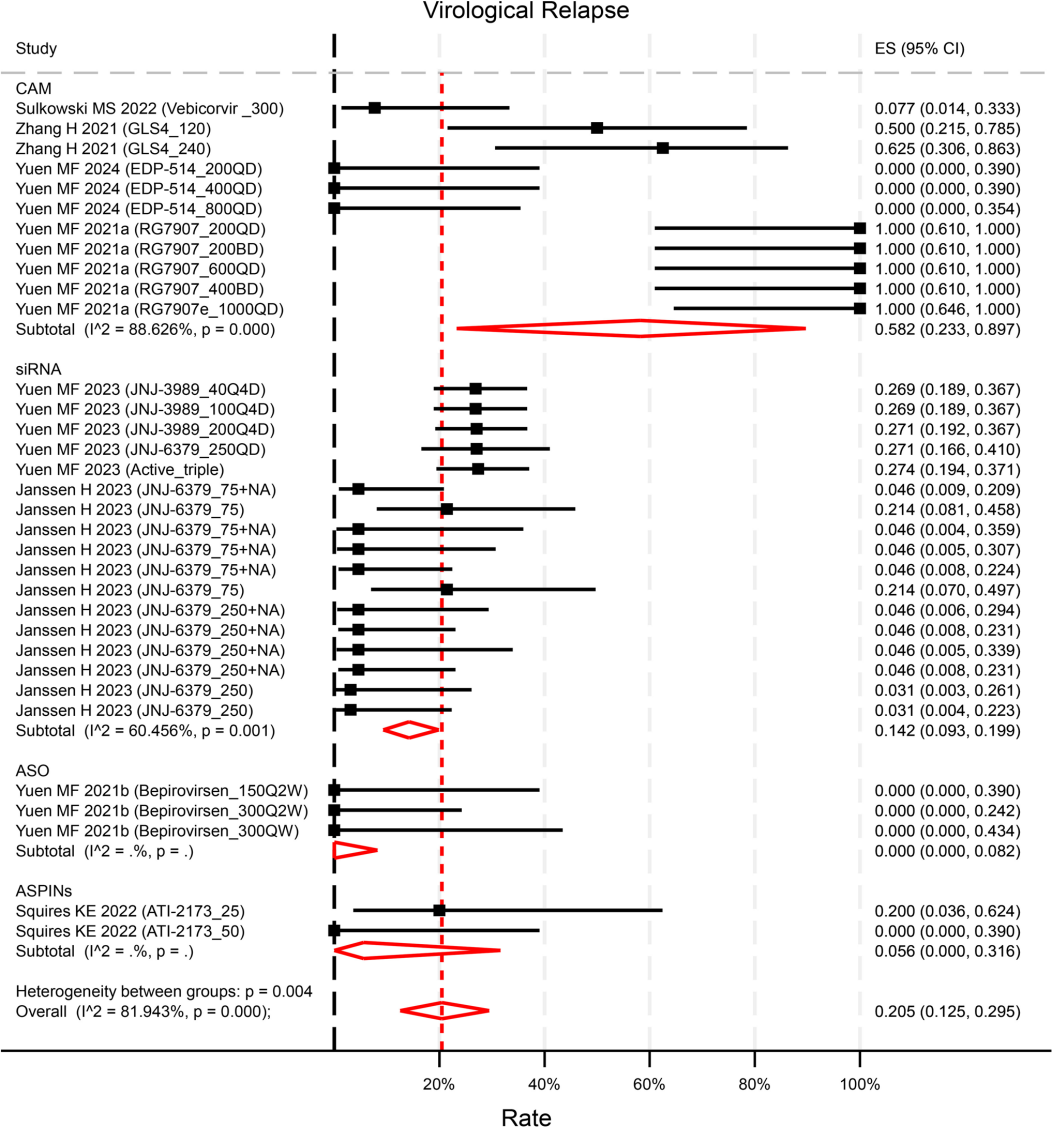
Reported Virological Relapse by compounds categories. The rate of VR in ASO and siRNA was statistically significantly lower than that in CAM (p = 0.031 and p = 0.011 respectively). p values were estimated by meta regression

Eight studies (contributing to 33 unique treatment arms of varying test doses) reported the virological relapse (VR) EOF (defined as HBV DNA rebound with different levels in each study). VR rates ranged widely from 0 to 100%. The pooled rate of VR was 20.5% (95%CI 12.5–29.5%, p < 0.001, I^2^ = 81.9%) (Fig. 5). The rate of VR in ASOs and siRNA was statistically significantly lower than that in capsid assembly modulators (CAMs) (p = 0.031 and p = 0.011 respectively). Most studies did not specify treatment for VR with only one study reporting retreatment with registered NAs for those experiencing VR. Compared with NA-treated control groups (5 studies contributing to 23 unique treatment arms), new therapies did not increase the risk of VR (OR = 1.32, 95%CI 0.73–2.37, p = 0.35) (Figure S10). However, compared to placebo control groups (2 studies contributing to 8 unique treatment arms), new therapies significantly increased the risk of VR (OR = 9.85, 95%CI 1.70–57.22, p = 0.01), though it was subject to a small sample size (Figure S10). The novel treatments demonstrated overall good tolerability, with no reported deaths related to the drugs across the trials. The rates of Adverse Events (AE) and Serious Adverse Events (SAE) were reported at 69.6% (95%CI 65.0–74.1%, p < 0.001, I^2^ = 57.2%, Figure S11) and 0.4% (95%CI 0–1.1%, p = 0.012, Figure S12) respectively. There was a higher risk of AEs observed in the treatment groups compared to NA-treated control groups (OR = 1.77, 95%CI 1.26–2.48, p < 0.001), but not compared to placebo control groups (OR = 1.02, 95%CI 0.58–1.79, p = 0.93, Figure S13). The risk of SAEs was not increased in treatment groups compared neither to NA-treated nor placebo control groups (Figure S14).

No evidence of publication bias (assessed through statistical funnel-plot asymmetry) was identified for the majority of analyses (Figure S15).

### Emerging barriers to functional cure

Our systematic review identified several barriers to achieving a functional cure with novel treatments. Despite all trials being in phases 1 and 2, their designs were complex. Five of the 19 trials had more than eight arms, with one trial having over 20 treatment arms [23]. Additionally, ten of the 19 trials lacked comparable groups to existing registered therapies. Follow-up durations were generally short, with a median time of 24 weeks (range: 4–48 weeks). Furthermore, unknown confounding factors appeared to impact therapeutic outcomes. In the B-clear trial for ASO [24], bepivrovirsen, it was demonstrated that there is a 9%–10% success rate in achieving sustained (24-week post-treatment) loss of HBsAg and HBV DNA in participants with CHB after a 24-week treatment with 300 mg bepirovirsen. This multicenter, investigator-unblinded, randomized phase 2b trial, positioning bepirovirsen as the sole investigational HBV drug advancing to phase 3, faces challenges due to unresolved dose-dependent, rebound, and unknown confounding issues. The phase 2b trial, following a phase 2a trial [25], failed to replicate the clear dose-dependent response observed earlier, introducing inconsistencies in the perceived efficacy of bepivrovirsen. Notably, group 1 (300 mg for 24 weeks) did not outperform group 2 (300 mg for 12 weeks + 150 mg for 12 weeks) in primary-outcome and HBsAg reduction for participants on NAs. Further, group 4 (300 mg without loading doses for 12 weeks) appeared more effective, achieving a 13% primary outcome and 43% HBsAg reduction ≥ 3 log10 IU/mL at the EOT, compared to 9% and 16% in counterpart group 3 with loading doses (Figure S10 and S11 in Yuen MF [24]). Further clarification is needed on evidence regarding dose-dependency. Additionally, significant rebound was observed, with proportions of primary-outcome occurrence declining from 25%, 16%, 9%, and 13% of patients on NA in groups 1, 2, 3, and 4, respectively at EOT to 9%, 9%, 3%, and 0% at end of follow-up. This observed rebound, in both NA and non-NA groups, suggests potential limitations in achieving a true functional cure compared to NA-treated patients. In the B-clear trial where identical treatment was administrated to group 1, 2, and 3 for the first 12 weeks, a comparable response was expected for three groups at the end of week 12. Unexpectedly, a higher proportion of on-NA participants in groups 1 (34%) and 2 (37%) achieved an HBsAg reduction ≥ 3 log10 IU/mL at week 12 compared to group 3 (16%), with recalculated proportions of HBsAg < LLOQ showing 18%, 20%, and 10% for groups 1, 2, and 3, respectively (Table S2). The difference in mean HBsAg levels also widened from week 8. Although not statistically significant when stratified by on-NA and not-on-NA, these findings suggest that there are potential unknown confounding factors that might influence treatment outcomes that require identifications. After excluding this study from our current meta-analysis, the pooled HBsAg loss at EOT and EOF was reduced to 0% (95%CI 0–0%), respectively (Figure S16).

## Discussion

This systematic review showed that the novel antiviral treatments have limited efficacy in achieving the desired therapeutic endpoints, including functional cure, HBsAg loss and reduction. The novel finite treatments did not demonstrate superiority over NA-treated control groups. The efficacy on HBsAg loss and reduction was generally not durable. Categories of anti-viral treatment in the studies, baseline HBsAg level of the enrolled subjects and unknown confounding factors had impact on achieving the therapeutic endpoints. Given the suboptimal efficacy shown in this meta-analysis, it will be challenging to reduce liver mortality to meet the WHO’s 2030 HBV elimination targets, particularly those related to achieving a functional cure.

Functional cure, which requires sustained HBsAg loss after treatment cessation, is currently the optimal treatment endpoint for CHB infection [26]. Achieving HBsAg loss with existing antiviral therapies is relatively rare, influenced by factors such as the patient’s age, duration of the infection, baseline viral load, the severity of liver disease and obesity. Nonetheless, when durable HBsAg loss is achieved by existing NA or IFN based antiviral therapies, it could represent a significant restoration of the host immune response [27]. This durable immune restoration is associated with a much lower risk of developing CHB-related liver complications such as liver cirrhosis and hepatocellular carcinoma. Achieving durable HBsAg loss, therefore, not only signifies viral control but also translates into better long-term health outcomes for patients.

In contrast, novel therapies, regardless of mechanism and target, have yet to achieve a clinical meaningful and durable HBsAg loss or HBsAg decline. Our meta-analysis revealed a minimal HBsAg loss at the end of treatment (0.9%) and end of follow-up (0.1%), with a notable rebound in HBsAg levels post-treatment (EOT: -1.20 log10 IU/mL; EOF: -0.64 log10 IU/mL). This rebound suggests that novel therapies might only inhibit HBsAg expression without engaging the immune system, unlike NA and pegylated interferons (pegIFN) which promote immune restoration [28]. Consequently, in the absence of durable HBsAg loss, novel therapies do not outperform existing NA treatments, indicating the need for continued optimization of current therapies and a deeper understanding of these mechanisms to advance future treatment options.

As our meta-analysis showed, compared to NA-treated control group the novel antiviral therapies did not significantly enhance the rates of functional cure (OR = 1.3 [95%CI 0.6–3.1], HBsAg loss at EOT (OR = 1.0, [95%CI 0.6–1.8]) or HBsAg loss at EOF (OR = 1.3, 95%CI 0.6–2.8). Unfortunately, none of the trials for novel therapies used pegIFN as the comparator, which preventing a direct comparison in our meta-analysis between pegIFN (alone or in combination with NAs) and novel therapies. Previous meta-analyses have shown that both pegIFN add-on to NAs and switching from NAs to pegIFN improve HBsAg loss compared to NA monotherapy, with the switch strategy being more effective [29, 30]. A recent randomized controlled trial further confirmed the superior efficacy of pegIFN-based therapies, reporting HBsAg loss rates of 12.9% and 12.1% in the pegIFN add-on and switch groups, respectively, among HBeAg-negative patients, compared to 0% in the NA monotherapy group [31]. Additionally, a recently published trial on the small interfering RNA (siRNA) compound Xalnesiran demonstrated that Xalnesiran in combination with pegIFN achieved the highest HBsAg loss rate (23%) among CHB patients compared to Xalnesiran alone or Xalnesiran with the toll-like receptor 7 agonist ruzotolimod [32]. However, this trial also lacked a pegIFN comparator arm (alone or in combination with NAs), limiting direct comparisons. These findings align with prior research highlighting pegIFN’s immunomodulatory effects, particularly its ability to enhance innate and adaptive immune responses—key mechanisms for achieving sustained viral suppression and functional cure [33]. Future trials should include a control arm with pegIFN to enable direct comparisons of efficacy.

The major barriers to HBV cure include the persistent reservoirs for HBV replication and antigen production by covalently closed circular DNA (cccDNA) and integrated HBV DNA, the high viral burden (HBV DNA and HBsAg) and the impaired host innate and adaptive immune responses against HBV. From in-vitro and in-vivo studies, durable loss of HBsAg requires restoration of effective innate and adoptive host immunity against hepatitis B virus [34]. This is demonstrated in the setting of allogeneic bone marrow transplantation where adoptive transfer of HLA-matched donor marrow with natural immunity against HBV can effectively lead to loss of HBsAg in CHB recipients [35]. Such loss of HBsAg has been correlated with the restoration of host innate and multi-specific T cell responses against HBV infection. Novel antiviral strategies that inhibit viral entry, viral translation and secretion of HBsAg, modulate capsid assembly, or target cccDNA transcription/degradation have shown promise in clinical trials [36, 37]. Novel immunomodulatory approaches including checkpoint inhibitors, metabolic modulation of T cells, therapeutic vaccines, adoptive transfer of genetically engineered T cells, and stimulation of innate and B-cell immune responses are being explored [38]. As yet, it is not fully understood how these novel therapies impact restoration of host immunity against HBV.

The presence of unknown confounding factors has emerged as a significant new barrier in the pursuit of a functional cure for HBV. Our systematic review and meta-analysis have shed light on these previously unidentified obstacles hindering the development of effective therapies. Specifically, the complicated trial designs, lack of comparable groups to existing registered therapies, and short follow-up durations have obscured the accurate assessment of efficacy for these new therapeutic candidates. The trial of the ASO compound [24], bepivrovirsen, which has progressed to the critical Phase 3 stage, serves as a prime example of the impact of these unknown confounding factors. Despite the identical initial 12 weeks of treatment period, the trial data revealed significant differences in the treatment effects during this period. This observation underscores the need for a deeper understanding of the complex interplay of factors that can influence the outcomes of HBV therapies. Identifying these barriers may shed light on how to improve the effectiveness of novel therapy and design better future studies.

Our study has limitations. The search was restricted to specific databases, which may have resulted in the omission of relevant studies. Although we attempted to mitigate this by comparing our search with existing review papers and the grey literature, the exclusion of certain compounds, such as bulevirtide—primarily indicated for hepatitis B and hepatitis D virus coinfection—limits the comprehensiveness of our analysis. Future reviews focused on specific populations, such as those with HBV and HCV coinfection or HBV and HDV coinfection, are warranted. Moreover, inconsistent outcome reporting and reliance on published data without access to raw data limits the analysis despite our efforts to standardize data for synthesis. Additionally, the varied follow-up durations raise concerns about long-term efficacy and safety.

## Conclusion

The achievement of a functional cure for HBV through current treatments is rare but durable once attained. Novel antiviral treatments, while targeting functional cure, often fall short in achieving this desired clinical endpoint. It is imperative to reconsider investment in the development of new antiviral drugs and to prioritize funding for proven effective public health initiatives such as universal vaccination, universal screening, and timely linkage to treatment. These initiatives hold the potential to significantly reduce the disease burden caused by HBV infection worldwide.

## Supplementary Information

Below is the link to the electronic supplementary material.

Supplementary file1 (DOCX 869 KB)

Supplementary file2 (DOCX 31 KB)

## Data Availability

All data will be shared upon request to the corresponding author.

## Abbreviations

CHB: Chronic hepatitis B
HCC: Hepatocellular carcinoma
HBV: Hepatitis B virus
WHO: The World Health Organization
HBsAg: Hepatitis B surface antigen
PEG-IFNa: Pegylated-interferon-a
pegIFN: Pegylate interferons
NAs: Nucleos(t)ide analogues
EOT: End of treatment
EOF: End of follow-up
AE: Adverse events
SAE: Serious adverse events
LLOQ: Lower limit of quantitation
siRNA: Small interfering RNA
ASOs: Antisense oligonucleotides
VR: Virological relapse
CAMs: Capsid assembly modulators
