# Supplementary material for: Functional cure with new antiviral therapy for hepatitis B virus: a systematic review and meta-analysis
Source: Hepatol Int. 2025 Jun 18;19(4):773–95. doi: 10.1007/s12072-025-10823-5 (PMC12287141; doi:10.1007/s12072-025-10823-5)
Supplement: Supplementary file 1 — Supplementary file1 (DOCX 869 KB) [file 12072_2025_10823_MOESM1_ESM.docx]

**Supplementary Materials**

**Supplementary Section 1: Search on ClinicalTrials.gov (**[**https://clinicaltrials.gov/**](https://clinicaltrials.gov/)**)**

We used the search box provided on the web to identify recently registered trials for Hepatitis B virus. In the Condition/disease box, we used “Chronic Hepatitis B” and “Hepatitis B” as provided by the search box. We limited the search to “Phase 1 to Phase 3”, “Interventional” study, and posted from Jan 1 2018 to Dec 31 2023. The search using “Chronic Hepatitis B” and its synonyms of conditions or diseases generated 167 records while “Hepatitis B” and its synonyms generated 244 records. Merging two search and removing duplicative records resulted a total of 244 unique records.

We excluded 36 and 49 records investigating vaccines and non-anti-viral therapies in CHB respectively; 14 records using registered drugs or prodrug of registered drugs; and 55 records investigating treatments for cirrhosis, HCC, other complications due to HBV and HBV coinfected with HDV, HIV, and HCV. It remained 90 records corresponding to 42 unique compounds (appendix table 1).

We searched these 42 unique compounds one by one in PubMed and Google to identify published full articles. Excluding 30 unique compounds with no published full articles in CHB patients, and including 3 compounds identified from review papers and grey literature, we landed on 19 full articles corresponding to 15 unique compounds.

| Compounds | Category | Records | Status | | | | | Number of articles | Included in meta |
| --- | --- | --- | --- | --- | --- | --- | --- | --- | --- |
|  |  |  | **Completed** | **Recruiting** | **Not recruiting** | **Terminated** | **Suspended** |  |  |
| JNJ-73763989/JNJ-56136379 | siRNA | 8 | 3 (2 Phase II, 1 Phase I) |  | 4 (2 Phase II, 2 Phase I) | 1 (Phase II) |  | 3 | Jassen 2023, Yuen MF 2023 |
| Vebicorvir (ABI-H0731) | CAM | 6 | 3 (2 Phase I, 1 Phase II) |  |  | 3 (Phase I) |  | 5 | Yuen MF 2022, Sulkowski MS 2022 |
| Bepirovirsen | ASO | 9 | 3 (2 Phase I, 1 Phase II) | 3 (2 Phase II, 1 Phase I) | 3 (2 Phase II, 1 Phase I) |  |  | 4 | Yuen MF 2022, Yuen MF 2021 |
| ZM-H1505R | CAM | 2 | 1 (Phase II) | 1 (Phase I/II) |  |  |  | 1 | Jia HY 2023 |
| ABI-H2158 | CAM | 4 | 3 (2 Phase I, 1 Phase II) |  |  | 1 (Phase II/III) |  | 2 | Agarwal K 2023 |
| VIR-2218 | siRNA | 8 | 3 (Phase II) | 2 (1 Phase I, 1 Phase II) | 3 (Phase II) |  |  | 3 | Gane EJ 2023 |
| ATI-2173 | ASPINs | 4 | 1 (Phase I) |  |  | 3 (2 Phase II, 1 Phase I) |  | 3 | Squires KE 2023 |
| EDP-514 | CAM | 3 | 3 (2 Phase I, 1 Phase II) |  |  |  |  | 2 | Yuen MF 2024, Feld JJ 2022 |
| GLS4 | CAM | 3 | 3 (2 Phase I, 1 Phase II) |  |  |  |  | 10 | Zhang H 2021 |
| RO7049389/RO7445482 (RG6346) | CAM/siRNA | 2 | 1 (Phase II) |  | 1 (Phase II) |  |  | 6 | Gane EJ 2023, Yuen MF 2021 |
| RO7062931 | ASO | 1 | 1 (Phase I) |  |  |  |  | 2 | Gane EJ 2021 |
| AB-506 | CAM | NR | Phase Ia/Ib |  |  |  |  | 4 | Yuen MF 2022 |
| GSK3389404 | ASO | 1 | Phase IIa |  |  |  |  | 1 | Yuen MF 2022 |
|  |  |  |  |  |  |  |  |  |  |
| CRV431 | Cyclophilin inhibitors | 1 | 1 (Phase I) |  |  |  |  | 3 | Preclinical |
| GST-HG131 | CAM | 1 |  |  |  |  |  | 2 | Preclinical/Healthy subjects |
| RBD1016 | siRNA | 2 | 1 (Phase I/II) | 1 (Phase I) |  |  |  | 1 | None human |
| AB-729 | siRNA | 2 |  |  | 2 (1 Phase I, 1 Phase II) |  |  | 1 | None human |
| QL-007 | CAM | 3 |  |  |  |  |  | 1 | None human |
| APG-1387 | IAP | 2 | 1 (Phase II) | 1 (Phase II) |  |  |  | 1 | None human |
| PA1010 | NAPs | 1 | 1 (Phase I) |  |  |  |  | 0 |  |
| Freethiadine | CAM | 1 | 1 (Phase I) |  |  |  |  | 0 |  |
| ABI-H3733 | CAM | 2 | 2 (1 Phase I, 1 Phase II) |  |  |  |  | 0 |  |
| HEC121120 | CAM | 1 |  |  |  |  | 1 (Phase II) | 0 |  |
| ABI-4334 | CAM | 1 | 1 (Phase I) |  |  |  |  | 0 |  |
| EDP-721 | HBV RNA Destabilizer | 1 |  |  |  | 1 (Phase II) |  | 0 |  |
| ALG-020572 | ASO | 1 |  |  |  | 1 (Phase III) |  | 0 |  |
| ALG-010133 | NAPs | 1 |  |  |  | 1 (Phase I) |  | 0 |  |
| DCR-HBVS | siRNA | 1 | 1 (Phase I) |  |  |  |  | 0 |  |
| DA-2803 | Anti-viral | 1 | 1 (Phase II) |  |  |  |  | 0 |  |
| RO7239958 | LNAs | 1 |  |  |  | 1 (Phase I) |  | 0 |  |
| JNJ-440 | CAM | 1 | 1 (Phase II) |  |  |  |  | 0 |  |
| JNJ-64457744 | siRNA | 1 |  |  |  | 1 (Phase II) |  | 0 |  |
| GST-HG141 | CAM | 3 | 1 (Phase I) | 1 (Phase I) |  |  |  | 0 |  |
| GSK3965193 | PAPD5/7 Inhibitor | 1 |  | 1 (Phase I) |  |  |  | 0 |  |
| Hepalatide | Entry inhibitor | 2 |  | 2 (1 Phase II, 1 Phase I) |  |  |  | 0 |  |
| ALG-000184 | CAM | 1 |  | 1 (Phase II) |  |  |  | 0 |  |
| AHB-137 | ASO | 2 |  | 2 (Phase II) |  |  |  | 0 |  |
| STSG-0002 | siRNA | 2 |  | 2 (1 Phase I, 1 Phase II) |  |  |  | 0 |  |
| LP-128 | NAPs | 1 |  | 1 (Phase I) |  |  |  | 0 |  |
| TQA3038 | siRNA | 1 |  |  | 1 (Phase I) |  |  | 0 |  |
| TQA3605 | CAM | 1 |  | 1 (Phase I/II) |  |  |  | 0 |  |
| ALG-125755 | siRNA | 1 |  |  | 1 (Phase II) |  |  | 0 |  |
| HRS-5635 | HBV RNA inhibitor | 1 |  | 1 (Phase II) |  |  |  | 0 |  |
| Table S1. Summary of the compounds identified | | | | | | | | | |

**Supplementary Section 2: Data standardization -** Imputation of missing SDs

A total of 17 studies with 73 unique treatment arms reported HBsAg changes from baseline at EOT. Three studies with 9 unique treatment arms had missing SD for the reported mean change of HBsAg. It was a relatively small number of treatment arms (9/73=12.3%). Thus, we imputed the missing SDs for each arm by calculating the pooled SD from all the other studies in our meta-analysis using the formula below:


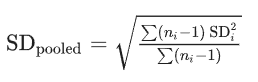


The imputed SD was transformed to SE using SE = SD/√(sample size) when needed.

ANOVA intraclass correlation coefficient (ICC) was used to compare the effect sizes of trials with reported SDs against the hypothetical effect sizes of the same trials based on the imputed SDs to examine the appropriateness of the imputation. SDs was needed in the comparisons between treatment and control groups in changes of HBsAg level at EOT in our meta-analysis. Thus, we used standardized mean difference (SMD) from above analysis to examine the appropriateness of the imputation. By replacing the SD for each trial by the pooled SD from all the other trials in our meta-analysis, the agreement between actual SMDs and imputed SMDs was 0.61 (95%CI 0.22 – 0.83), which indicates a good agreement. The imputed SDs could be deemed as appropriate to use in our meta-analysis.

**Supplementary Sections 3: Results from quantitative synthesis**

**Figure S1. Effect of Baseline HBsAg level on reported rate of functional cure.** Study n = 7 contributing to 31 unique treatment arms; Baseline HBsAg level did not have an impact on reported rate of functional cure with a coefficient of -0.07 and p=0.56. Coefficient and p value was estimated by meta regression.

**Figure S2. Comparison between treatment and NA-treated control groups in achieving functional cure.** Effect size was shown as log odds ratio. Compared to NA-treated control groups, the novel antiviral treatment did not significantly enhance the rate of functional cure (OR = exp(0.297) = 1.346, 95%CI 0.592 – 3.062).

**Figure S3. Effect of Baseline HBsAg level on reported rate of HBsAg at EOT.** Study n = 8 contributing to 42 unique treatment arms; Baseline HBsAg level did not have an impact on reported rate of HBsAg loss at EOT with a coefficient of -0.03 and p=0.38. Coefficient and p value was estimated by meta regression.

**A**

**B**

**Figure S4. Comparison in rate of HBsAg loss at (A) EOT and (B) EOF between treatment and NA-treated control groups.** Effect size was shown as log odds ratio. Compared to NA-treated control groups, the novel antiviral treatment did not significantly enhance the rate at EOT (OR = exp(0.019) = 1·019, 95%CI 0·573-1·813, p=0·94) or EOF (OR=exp(0.232) = 1·261, 95%CI 0·571-2·784, p=0·56)

**Figure S5. Effect of Baseline HBsAg level on HBsAg change from baseline measured at EOT.** Study n = 17 contributing to 73 unique treatment arms; Low baseline HBsAg favors EOT HBsAg decline (p=0.008). Coefficient and p value was estimated by meta regression.

**A**

**B**

**Figure S6. Comparison in changes of HBsAg level from baseline at EOT between treatment and (A) NA-treated control, (B) placebo control groups.** Effect size was shown as Hedges’s g. Compared to NA-treated control groups, the novel antiviral treatment did not induce a significantly larger decline in HBsAg EOT (Standardized Mean Difference [SMD] =-0.25, 95%CI -0.89 to 0.39, p=0.44, I^2^=90.3%). Compared to placebo control, novel treatment significantly induced a larger decline in HBsAg EOT (SMD = -0.35, 95%CI -0.63 to -0.06, p=0.02, I2=14.4%)

**Figure S7. HBsAg change from baseline measured at EOT in those with reported decline (negative change).**

**A**

**B**

**Figure S8. HBsAg change from baseline measured at (A) EOT and (B) EOF in those with reported decline (negative change).** Among the five studies with both reported negative on-tx and off-tx change from baseline (contributing to 26 unique treatment arms), the pooled on-tx decline from baseline was -1.20 log10 IU/mL (95%CI -1.44 to -0.96, p<0.001, I^2^=99.5%) while the pooled off-tx decline from baseline was -0.64 log10 IU/mL (95%CI -0.73 to -0.56, p<0.001, I^2^=98.6%).

**Figure S9. Dose-response in HBsAg change from baseline measured at EOT.** Meta-regression showed that the higher the dose the smaller the decline (coefficient= 0.0000103, p=0.002, tau^2^=0.47, Adj R-squared=12.0%)

******Figure S10. Comparison in virological relapse between treatment and (A) NA-treated, (B) placebo control groups.** Effect size was shown as log odds ratio. Compared with NA-treated control groups (5 studies contributing to 23 unique treatment arms), new therapies did not increase the risk of VR (OR= 1.32, 95%CI 0.73-2.37, p=0.35). Compared to placebo control groups (2 studies contributing to 8 unique treatment arms), new therapies significantly increased the risk of VR (OR= 9.85, 95%CI 1.70-57.22, p=0.01).

**B**

**A**

**Figure S11. Reported rate of Adverse Events.**

**Figure S12. Reported rate of Serious Adverse Events.**

**A**

**B**

**Figure S13. Comparison in adverse events between treatment and (A) NA-treated, (B) placebo control groups.** Effect size was shown as log odds ratio. There was a higher risk of AE observed in the treatment groups compared to NA-treated control groups (OR=exp(0.572) = 1.772, 95%CI 1.264 – 2.484, p<0.001), but not compared to placebo control groups (OR=exp(0.024) = 1.024, 95%CI 0.587 – 1.788, p=0.93).

**A**

**B**

**Figure S14. Comparison in serious adverse events between treatment and (A) NA-treated, (B) placebo control groups.** The risk of SAE was not increased in treatment groups compared neither to NA-treated (OR=exp(0.662) = 1.932, 95%CI 0.881 – 4.263, p=0.09) nor placebo control groups (OR=exp(0.004) = 1.004, 95%CI 0.428 – 2.358, p=0.99).

**Figure S15. Dio plot to estimate the small study effect.**

|  | **HBsAg < LLOD (Proportion)** | | | **P value** | | |
| --- | --- | --- | --- | --- | --- | --- |
| **Trial groups** | **Group 1** | **Group 2** | **Group 3** | **1 vs.2** | **2 vs.3** | **1 vs.3** |
| On-NA | 18% (12/68) | 21% (14/68) | 10% (7/68) | 0.66 | 0.07 | 0.18 |
| Not-on-NA | 19% (13/70) | 19% (13/68) | 10% (7/68) | 0.99 | 0.14 | 0.13 |
| Both | 18% (25/138) | 20% (27/136) | 10% (14/136) | 0.20 | 0.02 | 0.057 |
| **Table S2 Response at week-12**  We recalculated the proportion of participants in group 1, 2, and 3 with HBsAg < LLOD over time using Figure 2 and Figure S7 from Yuen MF (NEJM 2022), shown in the table above. Overall, 18%, 20% and 10% participants in group 1, 2, and 3 respectively achieved HBsAg < LLOD at week 12 (p = 0.02 and 0.057 for comparison between group 2 and 3, and between group 1 and 3 respectively). | | | | | | |

**A**

**B**

**Figure S16. Reported rate of HBsAg loss at (A) EOT and (B) EOF excluding B-clear study.** The pooled rate of HBsAg loss at EOT and EOF was reduced to both 0% (95%CI 0-0%) from 0.9% (95%CI 0.0 – 3.1%, p=0.064, I2=67.1%) and 0.1% (95%CI 0 – 0.8%, p=0.36, I2=12.7%) respectively.
